# Gamma Aminobutyric Acidergic and Neuronal Structural Markers in the Nucleus Accumbens Core Underlie Trait-like Impulsive Behavior

**DOI:** 10.1016/j.biopsych.2013.07.013

**Published:** 2014-01-15

**Authors:** Daniele Caprioli, Stephen J. Sawiak, Emiliano Merlo, David E.H. Theobald, Marcia Spoelder, Bianca Jupp, Valerie Voon, T. Adrian Carpenter, Barry J. Everitt, Trevor W. Robbins, Jeffrey W. Dalley

**Affiliations:** a Behavioural and Clinical Neuroscience Institute and Department of Psychology, University of Cambridge, Addenbrooke’s Hospital, Cambridge, United Kingdom; b Wolfson Brain Imaging Centre, Department of Clinical Neurosciences, Addenbrooke’s Hospital, Cambridge, United Kingdom; c Department of Psychiatry, University of Cambridge, Addenbrooke’s Hospital, Cambridge, United Kingdom; d Division Neurobiology of Behaviour, Department of Animals in Science and Society, Utrecht University, Utrecht, The Netherlands; e Cambridgeshire and Peterborough National Health Service Foundation Trust, Cambridge, United Kingdom

**Keywords:** Attention-deficit/hyperactivity disorder, GABA, impulsivity, magnetic resonance imaging, nucleus accumbens, psychostimulants

## Abstract

**Background:**

Pathological forms of impulsivity are manifest in a number of psychiatric disorders listed in DSM-5, including attention-deficit/hyperactivity disorder and substance use disorder. However, the molecular and cellular substrates of impulsivity are poorly understood. Here, we investigated a specific form of motor impulsivity in rats, namely premature responding, on a five-choice serial reaction time task.

**Methods:**

We used in vivo voxel-based magnetic resonance imaging and ex vivo Western blot analyses to investigate putative structural, neuronal, and glial protein markers in low-impulsive (LI) and high-impulsive rats. We also investigated whether messenger RNA interference targeting glutamate decarboxylase 65/67 (GAD_65/67_) gene expression in the nucleus accumbens core (NAcbC) is sufficient to increase impulsivity in LI rats.

**Results:**

We identified structural and molecular abnormalities in the NAcbC associated with motor impulsivity in rats. We report a reduction in gray matter density in the left NAcbC of high-impulsive rats, with corresponding reductions in this region of glutamate decarboxylase (GAD_65/67_) and markers of dendritic spines and microtubules. We further demonstrate that the experimental reduction of de novo of GAD_65/67_ expression bilaterally in the NAcbC is sufficient to increase impulsivity in LI rats.

**Conclusions:**

These results reveal a novel mechanism of impulsivity in rats involving gamma aminobutyric acidergic and structural abnormalities in the NAcbC with potential relevance to the etiology and treatment of attention-deficit/hyperactivity disorder and related disorders.

The concept of impulsivity encompasses a wide variety of behaviors spanning a failure of motor inhibition to individual predisposition to choose small, immediate rewards as opposed to large but delayed rewards 1, 2. Deconstruction of this behavior reveals two main subgroups: 1) motor impulsivity, including motor response inhibition assessed by failure to stop an already executed response and the high occurrence of premature or anticipatory responses; and 2) decisional impulsivity, which includes delay discounting and reflection impulsivity, involving cognitive choice mechanisms and the tendency to make rapid decisions without adequate consideration of alternatives (1). High levels of impulsivity are reported in attention-deficit/hyperactivity disorder (ADHD), conduct disorder, antisocial behavior, and substance use disorder (3). Here, we focus on a specific form of motor impulsivity in rats, assessed by the number of anticipatory responses made before the onset of a visual target stimulus on a five-choice serial reaction time task (5-CSRTT) (1), a task recently validated in humans to assess impulsivity in substance addictions and binge-eating disorder (4).

The underlying mechanisms of impulsivity are not well understood but putatively involve deficiencies in norepinephrine and dopamine (DA) transmission 5, 6, 7, 8, together with functional abnormalities in the prefrontal cortex (PFC) and striatum 9, 10, 11, 12, 13, 14, 15. Research has implicated the nucleus accumbens (NAcb) as a key brain region involved in the expression of impulsive behavior 1, 16, a function postulated to involve glutamatergic inputs from the amygdala, hippocampus, midline thalamus, and PFC, together with DA inputs from the mesolimbic DA system (17) that impinge on its core (NAcbC) and shell (NAcbS) subterritories 1, 16. Synaptic integration in the NAcb is governed by convergent glutamatergic and dopaminergic afferents on medium-sized, densely spiny gamma aminobutyric acid (GABA)-ergic neurons to determine behavioral output 18, 19, 20. Medium-spiny neurons (MSNs) thus play a critical role in the integration and gating of synaptic transmission in the NAcb. Surprisingly, however, few studies have investigated their involvement in the expression of impulsive behavior.

High impulsivity on the 5-CSRTT is present in 8% to 14% of the Lister-hooded rat strain and persists throughout adulthood 21, 22. High-impulsive (HI) rats show escalation of intravenous cocaine and nicotine self-administration 21, 23, an increased propensity for relapse after abstinence, and compulsive drug taking 24, 25 compared with low-impulsive (LI) rats. High impulsivity on the 5-CSRTT is associated with reduced availability of DA D_2/3_ receptors in the ventral striatum (including the NAcb) but not the dorsal striatum 21, 26. In the present study, we extend these findings using in vivo magnetic resonance imaging (MRI) and ex vivo protein analysis to isolate structural and molecular biomarkers associated with high impulsivity in rats. We report that high impulsivity on the 5-CSRTT is associated with putative alterations in dendritic spine density and is selectively and causally determined by GABA-dependent mechanisms in the NAcbC.

## Methods and Materials

### Subjects

We screened a total of 240 Lister-hooded rats (Charles River, Kent, United Kingdom) for low and high impulsivity on the 5-CSRTT. We selected for the present study *n* = 6 HI rats, *n* = 43 LI rats, and *n* = 6 mid-impulsive (MI) rats. The larger number of LI rats reflects their use in the glutamate decarboxylase 65/67 (GAD_65/67_) antisense experiment described below. Surplus HI and MI rats were used for other studies. Subjects weighed 250 g to 275 g at the start of behavioral training and were housed in groups of four in humidity- and temperature-controlled holding rooms (22°C) under a reversed light/dark cycle (white lights off/red lights on from 7:30 am to 7:30 pm). Rats were mildly food restricted to no more than 85% of their free feeding weights and water was available ad libitum. Experimental procedures complied with the United Kingdom Animals (Scientific Procedures) Act of 1986 and local institutional ethical guidelines.

### Impulsivity Assessment

Details of the behavioral apparatus and training are provided in Supplement 1 and published elsewhere (27). Rats were trained on the 5-CSRTT to detect the location of brief visual stimuli (.7 sec) presented in a pseudorandom manner in one of five apertures. Correct responses were rewarded with a food pellet delivered in the magazine. Incorrect responses and omissions were signaled by the house light being extinguished for 5 seconds and no food delivery. A premature response was recorded if subjects responded before the onset of the stimulus and resulted in the same time-out period and loss of food reward as incorrect responses. Once rats had acquired the 5-CSRTT, they were ranked for impulsivity during a 3-week screening period. Each week consisted of 5 consecutive days of testing with days 1, 2, 4, and 5 comprising sessions each of 100 discrete trials with an intertrial interval (ITI) of 5 seconds (short ITI). During day 3, the ITI was increased to 7 seconds to increase the frequency of premature responses (long ITI). High-impulsive animals were defined as those making more than 50% of trials prematurely during each of three long ITI sessions. The lowest ranked animals were deemed LI, while rats with intermediate levels of impulsivity were deemed MI.

### Morphological Assessment by MRI

Magnetic resonance imaging scanning was carried out in HI, MI and LI rats (each group *n* = 6). Rats were anesthetized with 5% isoflurane and scanned in vivo using a 4.7T Bruker BioSpec 47/40 system (repetition time/effective echo time 3500/36 msec, echo train length 8, number of excitations 2, 256 × 256 × 96 field of view, 40 × 40 × 15 mm^3^, isotropic resolution 156 µm^3^). A 72-mm birdcage resonator was used for transmission and signals were detected with a 20 mm diameter surface coil (Supplement 1, Morphological Assessment by MRI).

### Data Processing

Our protocol for voxel-based morphometry was based on published methodology (28). Images were corrected for intensity nonhomogeneity due to the surface coil and then segmented into tissue maps corresponding to canonical gray matter (GM), white matter, and cerebrospinal fluid using SPM5 (29) (Wellcome Department of Clinical Neurology, London, United Kingdom; http://www.fil.ion.ucl.ac.uk) with the SPMMouse plugin (30). The resulting images were smoothed with an 800 µm isotropic Gaussian kernel using statistical parametric mapping and used as tissue probability maps in the unified segmentation algorithm (31).

Smoothed GM maps were fitted to a block design model to reveal differences between the LI, MI, and HI rats. A two-tailed Student *t* test was used to detect voxels where the mean GM signal differed between groups. The false discovery rate was controlled at a threshold positive false discovery rate <.05 as a control against multiple comparisons (32). The correlation between the GM score and impulsivity scores was determined by Pearson product-moment correlation coefficient (*r*). Williams test was used to evaluate the differences between the two dependent rho values (i.e., elements deriving from the same correlation matrix) calculated separately for the left and right hemispheres.

### Western Blot Analysis

One week after the completion of MRI scanning, HI and LI rats were sacrificed by carbon dioxide inhalation; thereafter, their brains were removed and snap-frozen at −80°C. Samples of the NAcbC and NAcbS, frontoparietal cortex, and caudate putamen (CPu) were microdissected with a .75 mm^2^ diameter punch from 1 mm sections of brain. Samples from one HI rat were lost during processing. Therefore, the final dataset for this aspect of the study contained *n* = 6 LI rats and *n* = 5 HI rats.

Immunodetection was performed using: 1) polyclonal rabbit anti-glial fibrillary acidic protein (Dako Cytomation, Glostrup, Denmark), a glial marker; 2) monoclonal mouse anti-Neuronal Nuclei (NeuN) (Millipore, Billerica, Massachusetts), a neuron-specific marker; 3) polyclonal rabbit anti-glutamate decarboxylase 65/67 (Millipore), the primary GABA synthesizing enzyme; 4) polyclonal rabbit anti-Neurabin II (Spinophilin; Sigma-Aldrich, St. Louis, Missouri), a dendritic spine marker; 5) monoclonal mouse anti-Microtubule Associated Protein 2 (MAP2) (Sigma), a marker for somatodendritic microtubule protein; and 6) monoclonal mouse anti-β-Actin (Abcam, United Kingdom), a housekeeping protein used as a loading control. Data analyses are described in Supplement 1, Western Blot Analysis.

### Antisense Oligodeoxynucleotides

Fully deprotected and desalted phosphorothioate oligodeoxynucleotides (ODNs), purified by polyacrylamide gel electrophoresis, were purchased from Sigma. Oligodeoxynucleotides were phosphorothioated on the three terminal bases of both 5’ and 3’ ends to increase stability and minimize nonspecific toxicity. Oligodeoxynucleotide sequences and concentrations were derived from previous studies 33, 34: glutamate decarboxylase 67 (GAD_67_) antisense oligonucleotide (ASO), glutamate decarboxylase 65 (GAD_65_) antisense, scrambled sequence control for GAD_67_, and scrambled sequence control for GAD_65_.

### Intracerebral Cannulation

Rats destined for the glutamate decarboxylase antisense experiments were ranked for low impulsivity as described above (*n* = 23). General anesthesia was induced with isoflurane (5%) and maintained throughout the surgery at 1.5% to 2% (flow rate, 2 L/min). Bilateral 22-gauge double-lumen guide cannulae (Plastics One, Sevenoaks, United Kingdom) were implanted above the NAcbC. Stereotaxic coordinates relative to bregma were: anterior-posterior +1.5 mm, medial-lateral ±1.9 mm, and dorsal-ventral −2.2 mm. Guide cannulae were occluded by a stylet and secured to the skull with dental cement and three stainless steel screws.

### Intracerebral ODN Administration

Infusions were given at 08:00, 8 hours before behavioral testing. Rats were then returned to their home cage until behavioral assessment on the 5-CSRTT. Injectors aimed at the NAcbC and CPu extended, respectively, 4.5 mm and 2 mm from the ventral tips of the guide cannulae. Infusions were made over 72 seconds (.3 µL per hemisphere) and based on a previous study containing 600 ng of either GAD_65/67_ antisense (ASO) or scramble (Scr) pairs 33, 34.

### Antisense Behavioral Procedure

Following surgery, LI rats were run on the 5-CSRTT (ITI = 5 sec) for 5 consecutive days. They were then challenged with three long intertrial interval sessions (ITI = 7 sec), each spaced 2 days apart, to obtain a stable level of premature responding. Rats were assigned to four groups matched for behavioral performance on the 5-CSRTT. The testing phase consisted of three long ITI sessions (ITI = 7 sec) spaced 2 days apart. On day 1, all rats received a bilateral infusion of phosphate-buffered saline (ODN vehicle) in the NAcbC. On day 2, one group received a bilateral infusion of GAD_65/67_ ASO, two groups received a unilateral infusion of GAD_65/67_ ASO (left or right, with Scr infused in the contralateral NAcbC), while the remaining group received Scr bilaterally in the NAcbC. On test day 3, rats that received a bilateral infusion of ASO or Scr were infused with phosphate-buffered saline to assess recovery. The remaining groups received a bilateral infusion of GAD_65/67_ ASO or Scr in the CPu. We validated the procedure in a separate group of selected LI rats (*n* = 14) to investigate the magnitude of reduction in GAD_65/67_ expression in the NAcb.

### Locomotor Activity

Spontaneous locomotor activity was assessed on the second test day, immediately after the completion of the 5-CSRTT behavioral session using six individual activity cages (20 × 25 × 20 cm). Each chamber contained two photocell beams located 1 cm above the floor and spaced evenly along the length of the cage. Two days before the locomotor activity assessment, rats were exposed to the chamber for 1 hour. A run was recorded if the two beams were broken within 200 milliseconds. Run data were collated into 18 × 5 minute bins.

### Histological Assessment of Cannulae Placement

At the completion of the experiment, rats were sacrificed with an intraperitoneal injection of sodium pentobarbital and perfused transcardially. Cannulae placements were verified under a light microscope and mapped onto published coronal sections of the rat brain (35).

## Results

### Stratification of Low and High Impulsive Rats

**Figure 1:**
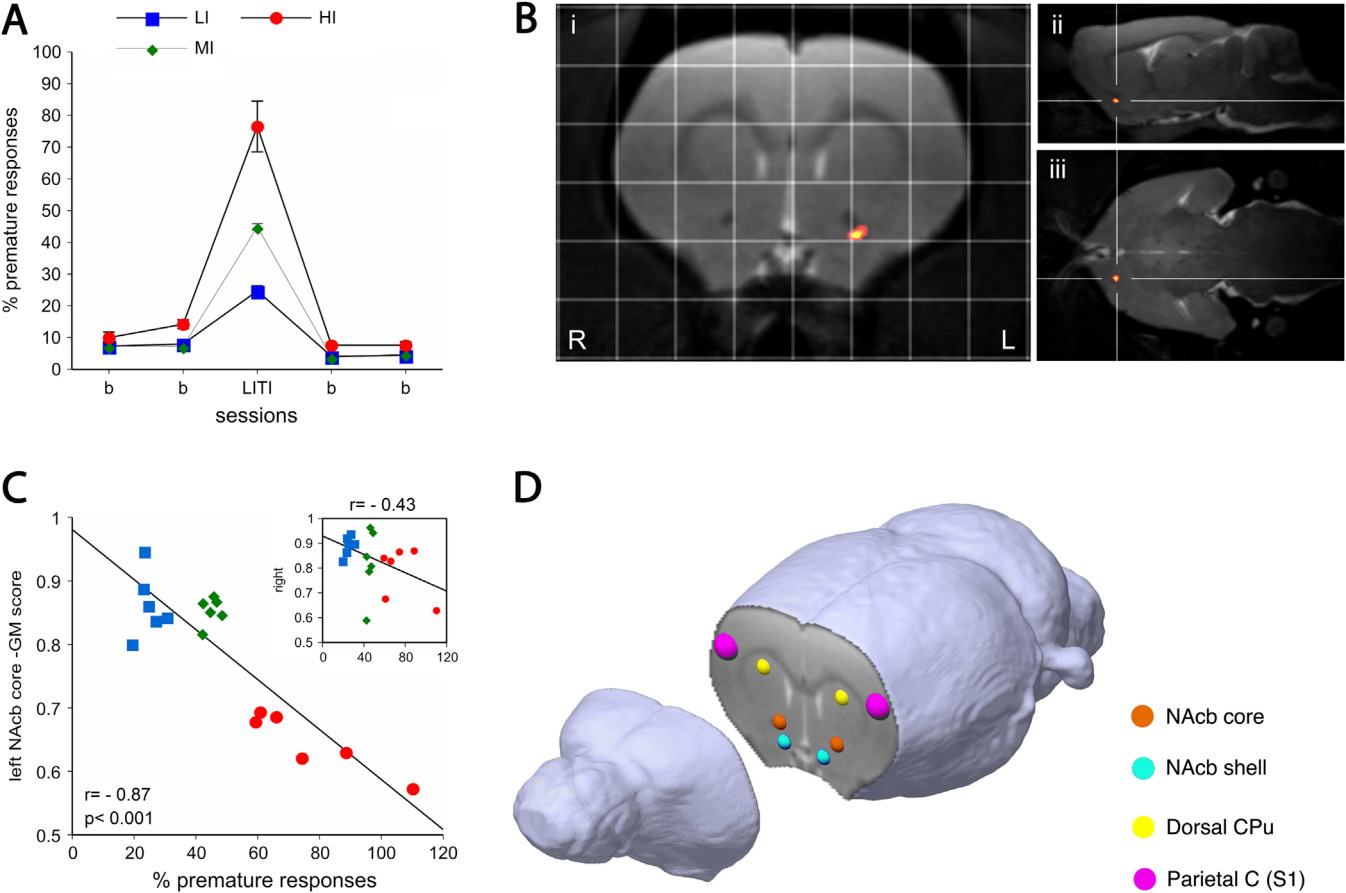
High impulsivity in rats is associated with a reduced density of gray matter (GM) in the left nucleus accumbens (NAcb) core. **(A)** High-impulsive rats (HI, *n* = 6) make more premature responses on the five-choice serial reaction time task compared with mid-impulsive rats (MI, *n* = 6) and low-impulsive rats (LI, *n* = 6) when the prestimulus waiting interval is increased to 7 seconds (long intertrial interval [LITI] = 7 sec) from the intervening 5-second interval (indicated by b). **(B)** Voxel-based morphometry analysis of orthogonal coronal (i), sagittal (ii), and horizontal (iii) sections superposed on an averaged magnetic resonance imaging template. The results indicate a significant reduction in the density of gray matter in the left NAcb core with a cluster extent of 29 voxels (uncorrected *F*_1,16_ = 116.2, *p*_family-wise error_ = .003), centered 2.3 mm anterior to bregma, 2.2 mm medial-lateral, 7.4 mm dorsal-ventral (35). **(C)** Negative correlation between impulsivity and GM scores, reported for individual subjects, of the most significant voxels in the left NAcb core (*r* = −.87, *p* < .001). The insert graph shows the corresponding, nonsignificant relationship between impulsivity and GM scores in the right NAcb core. **(D)** Three-dimensional composite image of the rat forebrain showing brain areas selected for Western blot analysis. C, cortex; CPu, caudate putamen; L, left; R, right.

Behavioral attributes of LI, MI, and HI rats on the 5-CSRTT are shown in Figure 1A and Table S1 in Supplement 1. We ranked and selected rats to form three groups based on the number of premature responses on the 5-CSRTT: HI rats that responded prematurely on more than 50% of trials (mean 76.6, *n* = 6); LI rats that were the lowest ranked animals (mean 24.7, *n* = 6); and MI rats that exhibited an intermediate level of impulsivity (mean 44.9, *n* = 6). With the exception of attentional accuracy, which showed a significant decrease in HI rats compared with LI and MI rats during the long ITI (HI vs. LI [*p* < .01]; HI vs. MI [*p* < .05])] but not during the shorter ITI (Table S1 in Supplement 1), no other behavioral variable was significantly affected in HI rats.

### MRI Localization of Highly Impulsive Behavior to the Left Nucleus Accumbens Core

We carried out a voxel-based morphological investigation of HI, MI, and LI rats using MRI. Three-dimensional reconstruction (Figure 1B) revealed a significantly reduced density of gray matter in the left NAcbC of HI rats (*p* < .05, false discovery rate corrected; HI vs. LI rats), which correlated inversely with the quantitative index of impulsivity on the 5-CSRTT (*p* < .001; *r* = −.87; Figure 1C). However, we found no significant correlation between gray matter score in the right NAcbC and impulsivity (Figure 1C insert). The lateralized relationship between gray matter density in the left NAcbC and impulsivity was confirmed by a significant pair-wise comparison between correlation coefficients for the left and right NAcbC (Williams test, *p* < .01).

### Highly Impulsive Behavior Is Associated with a Reduced Expression of Dendrite Spine Markers and GAD_65/67_ in the Left Nucleus Accumbens Core

**Figure 2:**
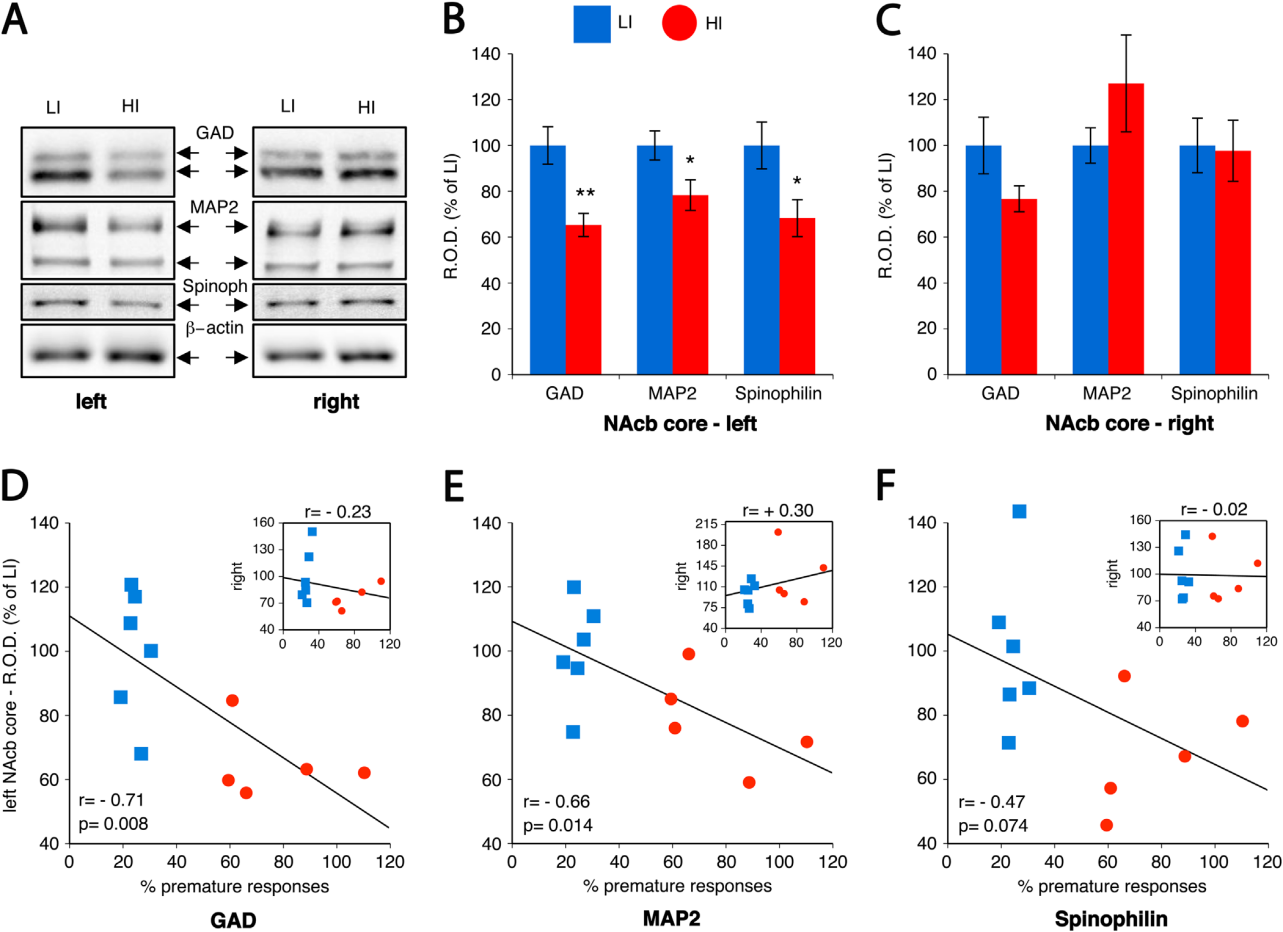
High impulsivity in rats is associated with a significant reduction in glutamate decarboxylase (GAD)_65/67_, microtubule-associated protein 2 (MAP2), and spinophilin (Spinoph) in the left nucleus accumbens (NAcb) core but not right NAcb core. **(A)** Representative immunoreactive bands from samples of the left and right NAcb core in low-impulsive (LI) and high-impulsive (HI) rats. **(B)** Densitometric quantification (relative optical density [R.O.D.] expressed as a % of the mean value of LI rats) of left NAcb core revealed a significant reduction of GAD_65/67_ (*t*_9_ = 3.48, **p < .01), MAP2 (*t*_9_ = 2.34, *p < .05), and spinophilin (*t*_9_ = 2.43, *p < .05) in HI rats compared with LI rats. Data are expressed as mean ± SEM. **(C)** Densitometric analysis of samples from the right NAcb core revealed no significant differences in GAD_65/67_, MAP2, and spinophilin (GAD_65/67_; *t*_9_ =1.66, *p* = .13). **(D–F)** Correlation between impulsivity scores and the relative optical density of GAD_65/67_ (**[D]***r* = −.71, *p* < .01), MAP2 (**[E]***r* = −.66, *p* < .05), and spinophilin (**[F]***r* = −.47, *p* = .074) in left NAcb core. Insert graph shows equivalent data for the right NAcb core.

We next used Western blot analysis to investigate structural, neuronal, and glial protein markers in the NAcbC, NAcbS, CPu, and frontoparietal cortex (Figure 1D) of the same HI and LI rats used above for MRI (Figure 2A). We found significantly lower levels of glutamate decarboxylase (GAD_65/67;_ *p* < .01, Figure 2B), as well as the dendritic marker microtubule associated protein (MAP2; *p* < .05, Figure 2B) and the dendritic spine marker spinophilin (*p* < .05, Figure 2B) in the left NAcbC of HI rats compared with LI rats. There were no significant differences in any of these markers in the right NAcbC, although there was a trend for GAD_65/67_ to be decreased in HI rats (*p* = .06, Figure 2C). We also identified a significant negative correlation between levels of GAD_65/67_ (*p* < .01; *r* = −.71; Figure 2D) and MAP2 (*p* < .05; *r* = −.66; Figure 2E) in the left NAcbC and impulsivity. Levels of spinophilin in the left NAcbC also showed a trend negative correlation with impulsivity (*p* = .074; *r* = −.47; Figure 2F). We found no differences between HI and LI rats in relation to a neuronal marker (NeuN) and a glial marker (glial fibrillary acidic protein) in the left or right NAcbC (Figure S1A in Supplement 1), NAcbS (Figure S1B in Supplement 1), CPu (Figure S1C in Supplement 1), or frontoparietal cortex (Figure S1D in Supplement 1). In addition, there was no significant difference between HI and LI rats in GAD_65/67_ content in the NAcbS (Figure S1B in Supplement 1), CPu (Figure S1C in Supplement 1), or frontoparietal cortex (Figure S1D in Supplement 1).

### Experimental Reduction of De Novo GAD_65/67_ Protein Expression in the Nucleus Accumbens Core Is Sufficient to Increase Impulsivity

**Figure 3:**
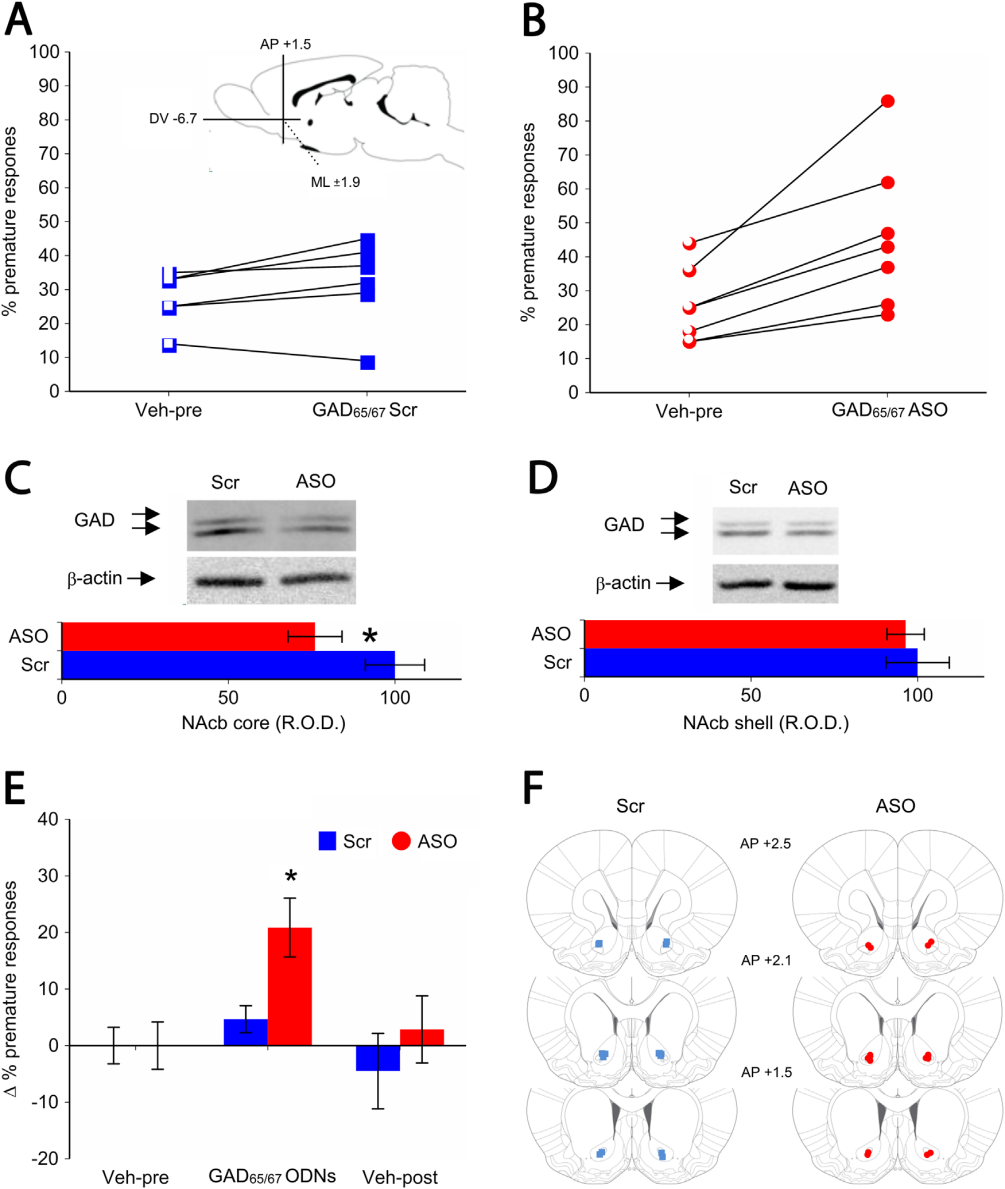
Bilateral reduction in glutamate decarboxylase 65/67 (GAD_65/67_) protein in the nucleus accumbens (NAcb) core increases impulsivity in low-impulsive rats on the five-choice serial reaction time task. **(A)** Individual responses of rats to GAD_65/67_ scrambled (Scr) sequence in the NAcb core showing no effect on premature responding compared with vehicle infusions in this region (*n* = 6). The insert graph shows the intended location of the oligodeoxynucleotide (ODN) microinfusions in the NAcb (35). **(B)** Individual responses of rats to GAD_65/67_ antisense in the NAcb core showing increased premature responding compared with vehicle infusions (*n* = 7). **(C)** Representative immunoblot and related densitometric analysis showing GAD_65/67_ antisense-induced decrease of GAD_65/67_ protein levels 8 hours after intra-NAcb core microinfusions in selected LI rats. *p < .05. **(D)** Representative immunoblot and related densitometric analysis of the adjacent NAcb shell showing no differences between GAD_65/67_ protein levels 8 hours intra-NAcb core microinfusions in low-impulsive rats. **(E)** Histograms show difference scores (± SEM) between the effects of vehicle infusions (pre-ODN and post-ODN) and ODN infusions (Scr and antisense oligonucleotide [ASO]). *p < .05 (Scr vs. ASO). **(F)** Injector tip locations in the NAcb core of rats injected with GAD_65/67_ Scr (left) and ASO (right). Anterior-posterior (AP) coordinates are relative to bregma (mm) (35). DV, dorsal ventral; ML, medial lateral; R.O.D., relative optical density; Vehicle-post/pre, GAD 65/67 ODNs exposure.

Finally, we investigated the effects of unilateral and bilateral intra-NAcbC microinfusions of GAD_65/67_ antisense oligodeoxynucleotides on impulsivity in LI rats (*n* = 23) (Tables S2 and S3 in Supplement 1). We found that GAD_65/67_ ASO resulted in a significant increase in impulsive responding in LI rats (*n* = 7; Figure 3B) compared with a second group of LI rats infused with a scrambled oligodeoxynucleotide sequence (Scr, *n* = 6; Figure 3A) (*p* < .05, Figure 3E). This effect was behaviorally selective with no significant effect of GAD_65/67_ ASO on locomotor activity or the speed and accuracy of responding on the 5-CSRTT (Table S2 in Supplement 1). Furthermore, we found no significant effect on impulsive responding or the speed and accuracy of responding on the 5-CSRTT, following a unilateral (left or right) microinfusion of GAD_65/67_ ASO in LI rats (Figure S2 and Table S3 in Supplement 1). We next injected GAD_65/67_ ASO or Scr bilaterally in the CPu. This intervention had no significant effect on impulsivity or the speed and accuracy of responding on the 5-CSRTT (Figure S2 and Table S3 in Supplement 1).

We validated the procedure in a separate group of selected LI rats (*n* = 14) to investigate the magnitude of reduction in GAD_65/67_ expression in the NAcbC. We observed a significant reduction of GAD_65/67_ protein levels after infusion of ASO compared with rats injected with Scr (*p* < .05) in the NAcbC (Figure 3C). However, we observed no significant effect of the ASO infusions on GAD_65/67_ expression in the NAcbS (Figure 3D), thus confirming the localization of the ASO infusions to the NAcbC.

## Discussion

We report a strong relationship between impulsivity on the 5-CSRTT and neuronal changes in the nucleus accumbens core, implicating alterations in GABA-containing neurons in this region. Our findings indicate a reduction in gray matter density in the NAcbC of HI rats, with corresponding reductions in this region of glutamate decarboxylase (GAD_65/67_), as well as dendritic spine and microtubule markers. We further demonstrate that the experimental intervention of reducing de novo GAD_65/67_ expression by infused antisense bilaterally in the NAcbC was sufficient to increase impulsivity, but not locomotor activity, in LI rats. These results suggest a novel mechanism of impulsivity in rats involving GABA-ergic dysfunction and putative alterations in the density of dendritic spines in the NAcbC with potential relevance to the etiology and treatment of ADHD and related disorders. Importantly, control manipulations confirmed that infusions of antisense in the NAcbC did not alter GAD_65/67_ levels in the NAcbS, while bilateral ASO infusions in the CPu had no significant effect on impulsivity. The close convergence in results between in vivo voxel-based MRI and ex vivo protein chemistry indicates putatively related abnormalities in the density and structure of dendrites and dendritic spines, especially in the left NAcbC. Our results suggest that high impulsivity may be caused by impaired synaptic integration of dopaminergic and glutamatergic afferents, specifically targeting the dendritic spines of GABA-ergic MSNs (36).

The present data add to growing evidence that impulsive responding on the 5-CSRTT can be modulated by interventions targeting the NAcbC. Thus, the effect of d-amphetamine to increase impulsivity on this task was blocked by intra-NAcbC infusions of the D_2/3_ receptor antagonist eticlopride (37). Impulsivity resulting from lesions of the PFC was also selectively blocked by intra-NAcbC infusions of the D_2/3_ receptor antagonist sulpiride (38).

The NAcbC also plays a key role in delay-discounting impulsivity. Thus, selective lesions of the NAcbC increased impulsive preference of rats for small, immediate rewards versus large but delayed rewards 16, 39, 40, 41, 42. Notably, rats selected for high impulsivity on the 5-CSRTT also showed steep discounting functions for delayed rewards (43). Furthermore, these studies are consistent with functional magnetic resonance imaging studies in humans showing a correlation of impulsive choice with ventral striatal/NAcb activity 44, 45 and altered ventral striatal activity in response to immediate and delayed rewards in patients with ADHD (46). Our molecular findings indicate, for the first time, that these impulsive responses may be modulated by an underlying impairment in GABA-ergic function in the NAcbC. However, further studies are needed to establish a role of GABA-ergic mechanisms in delay-discounting impulsivity as opposed to the form of impulsivity assessed in the present study.

While we did not observe differences in NeuN, suggesting that the number of neurons in the left NAcbC was unaltered in HI rats, the structural integrity and presumed density of dendritic spines in this region were severely affected and inversely predicted impulsivity on the 5-CSRTT. As dendritic spines represent the key loci of synaptic integration between excitatory glutamatergic projections from the PFC and dopaminergic inputs from the midbrain (47), these findings may suggest the NAcbC to be the neural locus of DA D_2/3_ receptor dysfunction in impulsive rats (21). However, a recent ex vivo autoradiography study from our group found that DA D_2/3_ receptors were reduced bilaterally in the NAcbS, not the NAcbC (26). This may be consistent with other evidence of opponent interactions between the NAcbS and the NAcbC 48, 49.

Recently, we reported a reduction in DA D_1_ receptors in the left NAcbC of HI rats compared with LI rats (26). Since DA D_1_ receptors are located postsynaptically on the dendrites of GABA-ergic MSNs, these results collectively support the hypothesis that dendritic spines may be reduced in density in the NAcbC of HI rats. Moreover, a reduction in DA D_1_ receptors in the left NAcbC may have been responsible for the observed reduction in GAD_65/67_ in this region. In support of this hypothesis, previous research has shown that intrastriatal administration of D_1_ receptor agonists increases GAD_65_ expression in striatal neurons 50, 51 and facilitates GABA release in the substantia nigra pars reticulata (52).

Our results indicate a strong inverse relationship between GAD_65/67_ and behavioral impulsivity (Figure 3). Although the reduction of GAD_65/67_ was striking in the left NAcbC, lower levels of GAD_65/67_ were also present in the right NAcbC of HI rats compared with LI rats (Figure 2C). This partial asymmetry in GAD_65/67_ levels between the left and right NAcbC of HI rats, in relation to LI rats, may explain why left-sided infusions of GAD_65/67_ ASO were insufficient to increase impulsivity in LI rats. Therefore, depletion of GAD_65/67_ in both the left and right NAcbC appears necessary for the expression of impulsivity. The origin of the hemispheric imbalance reported in the present study is unknown but may arise from genetic and/or environmental factors affecting trophic signals during development (53). Left/right asymmetries are often reported in rats 54, 55 and brain disorders, including ADHD 56, 57.

To date, there has been limited research on the role of GABA in impulsivity. However, male mice with a mutation to the fragile X mental retardation 1 gene (*Fmrl*) showed impaired attention and inhibitory response control, just like HI rats in the present study. Moreover, mutant *Fmrl* mice show a reduced expression of brain GAD_67_ 58, 59. In rats, inhibition of GABA synthesis in the PFC led to a marked increase in locomotor activity but did not affect visual attention on the 5-CSRTT (60). In the present study, inhibition of GAD_65/67_ expression in the NAcbC had no effect on either locomotor activity or visual attention but selectively increased impulsivity. Thus, our findings strongly indicate that trait-like impulsivity on the 5-CSRTT is linked to GABA dysfunction in the NAcbC. Although a recent autoradiography study found no abnormalities in benzodiazepine-sensitive GABA type A receptor binding in the NAcb of HI rats (26), a variety of benzodiazepine-insensitive GABA type A receptor subunits are expressed in this region (61), which may be subject to differential regulation and expression in highly impulsive rats. In light of the present findings, this possibility merits further investigation.

### Conclusions and Clinical Implications

The marked, mainly asymmetric decrease in gray matter and markers of GABA and dendritic function in the NAcbC suggest a novel mechanism underlying the etiology of a form of impulsivity linked to ADHD and comorbid disorders such as drug addiction.

Although the presumed genetic and environmental factors leading to the origin of this candidate neurobehavioral endophenotype require further investigation, our findings converge on the conclusion that GABA-related mechanisms may play a necessary role in the expression of impulsivity. Premature responding on the 5-CSRTT assesses several putative aspects of impulsivity, including timing, behavioral inhibition, and the capacity to tolerate delayed rewards (1). Rats exhibiting high impulsivity on this task are also delay averse and preferentially choose small, immediate rewards as opposed to large but delayed rewards (43). Clinically, an inability to delay gratification is strongly linked to alcoholism 62, 63, 64 cocaine and heroin addiction 65, 66, 67 and in rats is exacerbated by lesions of the NAcbC (39). A novel analogue of the rodent serial reaction time task has recently been developed with utility in human substance addictions and binge-eating disorder (4).

Our results not only confirm an important role for the NAcbC in a form of impulsivity indexed by the 5-CSRTT and delay discounting [previously described as waiting impulsivity (43)] but also begin to elucidate the underlying molecular and neuronal changes associated with this trait. Pathological abnormalities in the integrity of dendritic spines on MSNs in the NAcbC may be relevant for understanding why HI rats are predisposed to escalate nicotine and cocaine self-administration and to relapse after abstinence 21, 23, 24, 36, 68.

In addition, our molecular findings may be relevant to the mechanism of action of stimulant drugs such as methylphenidate and amphetamine in ADHD 1, 67. Medium-spiny neurons in the NAcbC are particularly sensitive to a decrease in spine density in the absence of DA (69). Since DA release is reportedly decreased in the NAcbC of impulsive rats on the 5-CSRTT (23), this may be a factor contributing to the hypothesized reduction in spine density on MSNs in the NAcbC of HI rats. Although this hypothesis requires confirmation using more direct techniques, for example, Golgi staining and the quantitative assessment of dendritic spine density in LI and HI rats, it is noteworthy that the stimulant drug cocaine increases dendritic spine density, especially in the NAcbC 70, 71. Thus, the clinical efficacy of stimulant drugs in ADHD may be mediated by dynamic molecular events that restore spine density on MSNs in the NAcbC.
