# Supplementary material for: Gamma Aminobutyric Acidergic and Neuronal Structural Markers in the Nucleus Accumbens Core Underlie Trait-like Impulsive Behavior
Source: Biol Psychiatry. 2014 Jan 15;75(2):115–23. doi: 10.1016/j.biopsych.2013.07.013 (PMC3898085; doi:10.1016/j.biopsych.2013.07.013)
Supplement: Supplementary file 1 — Supplementary Material [file mmc1.pdf]

Gamma-Aminobutyric Acid-ergic and Neuronal Structural Markers in the Nucleus Accumbens Core Underlie Trait-Like Impulsive Behavior

Supplemental Information

Subjects

We screened a total of 240 Lister-hooded rats (Charles River, UK) for low and high impulsivity on the 5-choice serial reaction time task (5-CSRTT). We selected for the present study in total n = 6 high-impulsive (HI) rats, n = 43 low-impulsive (LI) rats, and n = 6 mid-impulsive (MI) rats using a screening procedure described below. Subjects weighed 250-275 g at the start of behavioral training and were housed in groups of four in humidity and temperature-controlled holding rooms (22°C) under a reversed light/dark cycle (white lights off/red lights on from 07:30 to 19:30). Rats were mildly food restricted to no more than 85% of their free feeding weights and water was available *ad libitum.* Experimental procedures complied with the UK Animals (Scientific Procedures) Act of 1986.

Behavioral Apparatus and Training

Rats were trained in twelve 5-CSRTT chambers (30.5 x 24.1 x 29.2 cm; Med Associates, St. Albans, VT) (1). Each test chamber was enclosed in a ventilated wooden sound-attenuating cubicle and consisted of a curved wall containing five equally-spaced 2.5 cm square apertures equipped with infrared detectors and a 3W stimulus light at the rear of each aperture. A centrally-located magazine was located in the rear wall, which was equipped with an infrared detector, and connected to a food magazine which automatically delivered 45 mg reward pellets (Noyes dustless pellets, Research Diets, UK). The chambers were controlled by a PC using "Whisker server 2.8" and Five Choice client (version 2.6) (2).

Rats were trained over approximately 50 sessions to detect the location of a brief visual stimulus presented in a pseudo-random manner in one of the five apertures. Stimuli were presented every 5 s and correct responses were rewarded with a food pellet delivered in the magazine. Acquisition of the task was facilitated by the staged reduction in the duration of the target stimulus (ultimately to 0.7 s), as described previously (1). Animals were trained to a final stimulus duration of 0.7 s, not 0.5 s, as used in our previous experiments, because we used different 5-choice boxes in the present study (i.e., Med Associates Inc.), which incorporate dim LEDs compared with the more intense tungsten lamps used in older-style boxes (e.g., Paul Fray Ltd). Less intense stimuli require a longer stimulus-duration to ensure accuracy levels of approximately 75%.

Sessions consisted of 100 discrete trials. Individual trials were initiated by the rat entering the food magazine. At this point, the magazine light was extinguished and the inter-trial interval (ITI) of 5 s commenced. Rats were required to scan the 5 apertures and to withhold from responding until the onset of the stimulus. A correct response was recorded if subjects successfully detected the spatial location of the visual stimulus with a nose poke response. Correct trials were rewarded with a single 45 mg food pellet delivered to the food magazine. An incorrect response was recorded if subjects responded in a non-illuminated aperture. Incorrect trials were signaled by the house light being extinguished for 5 s and no food delivery. An omission was recorded if subjects failed to respond within 5 s of the onset of the target stimulus. Omissions were signaled in the same way as incorrect responses. A premature or impulsive response was recorded if subjects responded before the onset of the stimulus. Premature responses resulted in the same time-out period and loss of food reward as incorrect responses.

A number of behavioral parameters were measured on the 5-CSRTT: *choice accuracy,* %

correct responses / (correct + incorrect responses); *premature responding,* % premature responses / (correct + incorrect + omissions); o*missions*, % omissions / (correct + incorrect + omissions); *latency to make a correct response* after the onset of the target stimulus (ms); *latency to collect food* from the magazine after a correct trial (ms).

Once rats had acquired the 5-CSRTT (accuracy > 75%, omissions < 20% for one week) they were then ranked for impulsivity during a 3-week screening period. Each week consisted of five consecutive days of testing with days 1, 2, 4 and 5 comprising sessions of trials with an ITI of 5 s. During day 3, the ITI was increased to 7 s to increase the frequency of premature responses. The duration of this session was increased to 60 min to enable 100 trials to be completed. HI animals were defined as those making on average more than 50% of trials prematurely during the 3 long ITI (LITI) sessions. The lowest ranked animals were deemed LI whilst rats with intermediate levels of impulsivity were deemed MI. MI rats were used for the magnetic resonance imaging (MRI) study only.

Note that the absolute number of premature responses and % premature responses may differ if the number of trials completed is less than 100. In the present study all rats completed 100 trials with the exception of two high-impulsive rats that completed on average 96.1 ± 2.1% of trials during the short ITI sessions and 70.0 ± 3.0% during the long ITI. This reduction in the number of trials completed during the long ITI was due to a high number of premature responses (mean ± SEM/67.3 ± 2.2). This had no bearing on the selection of rats as LI, MI, or HI. Behavioral variables on the 5-CSRTT were analyzed by repeated measures analysis of variance (SPSS type III sum-of-squares method, version 17) to compare baseline and LITI performances between HI, MI and LI rats. Where applicable, violations of sphericity were corrected using Greenhouse-Geisser or Huynh-Feldt epsilons to adjust the degrees of freedom. Post-hoc tests

were carried out with Bonferroni's correction.

Morphological Assessment by MRI

MRI scanning was carried out in HI, MI and LI rats (each group n = 6). Rats were anesthetized with 5% isoflurane and transferred to the MRI suite. They were placed on a plastic sliding cradle equipped with ear bars and an incisor pipe bar that delivered the anesthetic agent. Rats were scanned *in vivo* using a 4.7T Bruker BioSpec 47/40 system (TR/TEeff 3500/36 ms, ETL 8, NEX 2, 256 × 256 × 96 FOV, 40 × 40 × 15 mm^3, isotropic resolution 156 μm^3). A 72 mm birdcage resonator was used for transmission and signals were detected with a 20 mm diameter surface coil.

Throughout the scanning procedure, isoflurane was maintained at 1.5-2% and the temperature monitored using a rectal thermometer and maintained to 37°C using a warm water blanket. Oxygen saturation, heart rate and breathing rate were strictly monitored and maintained within the normal range throughout the MRI scans, which each lasted 1 h 29 min. At the end of the MRI scan, rats were transferred to a recovery chamber and carefully monitored before being returned to the holding room.

Data Processing

Our protocol for voxel-based morphometry (VBM) was based on published methodology (3). Images were corrected for intensity non-homogeneity due to the surface coil and then segmented into tissue maps corresponding to canonical gray matter, white matter and cerebrospinal fluid (GM, WM and CSF, respectively) using SPM5 (4) (Wellcome Department of Clinical Neurology, London; http://www.fil.ion.ucl.ac.uk) with the SPMMouse plugin (5).

Tissue priors for this algorithm were generated as follows: an image was selected at random to act as a target for rigid alignment of all of the images. Images thus registered were averaged to produce a new target, used for affine registration of all of the images. The resulting images after affine transformation were averaged to produce a subsequent target, which was scaled by the inverse of the average scaling factor of each brain so as to have the mean size of all the brains. This image was segmented into GM, WM and CSF partitions using a k-means algorithm (6). The resulting images were smoothed with an 800 µm isotropic Gaussian kernel using SPM and used as tissue probability maps in the unified segmentation algorithm (7). Each brain was segmented using these maps as prior information. The resulting modulated maps from each brain were averaged to create a second set of average tissue maps. These maps formed the final priors used to segment each subject image in the unified segmentation algorithm to produce GM maps, which were used for VBM analysis. Maps were smoothed with an 800 µm isotropic Gaussian kernel. Throughout these pre-processing steps, the original images were used for every transformation to avoid accumulation of interpolation artifacts from successive steps.

The cluster used for the correlation analysis was automatically derived based on the significant voxels determined in the VBM analysis and fitted to a block design model to find differences between the LI, MI and HI rats. The corresponding area on the right side of the brain was chosen to mirror the same region on the left. A two-tailed Student's *t*-test was used to detect voxels where the mean GM signal differed between groups. The false-discovery rate (FDR) was controlled at a threshold $pFDR < 0.05$ as a control against multiple comparisons (8). The significant brain region was identified using surrounding structures as landmarks with reference to the histologically-based atlas of Paxinos and Watson (9). The correlation between the GM score (expected tissue volume fraction that is present in a voxel from a particular subject

adjusted by the local volume change in that voxel for that subject) and impulsivity scores was determined by Pearson's product-moment correlation coefficient (r). Williams test was used to evaluate the differences between the two dependent rho values (i.e., elements deriving from the same correlation matrix) calculated separately for the left and right hemisphere.

Western Blot Analysis

One week after the completion of MRI scanning, HI and LI rats were sacrificed by carbon dioxide inhalation followed by dislocation of the neck. Their brains were rapidly removed and snap frozen at -80°C. Samples of nucleus accumbens (NAcb) core and shell, fronto-parietal cortex, and caudate-putamen were micro-dissected with a 0.75 mm^2 diameter punch from 1 mm thick brain sections. Samples from one HI rat were lost during processing. Therefore, the final dataset for this aspect of the study contains n = 6 LI rats and n = 5 HI rats. The tissue punches were sonicated in Lysis buffer (Hepes 20 mM; pH 7.9; NaCl 150 mM; $MgCl_2$ 1 mM; EDTA 1 mM; DTT 1 mM; Pepstatin A 1 μg/ml; Leupeptin 10 μg/ml; PMSF 0,5 mM; Aprotinin 10 μg/ml) and centrifuged at 3000 *g* for 5 min. The supernatant (total proteins extract) was transferred to a fresh tube and stored at -80°C until use. Protein content in each extract was determined using a Nanodrop ND-1000 (Thermo Scientific, USA). Ten micrograms of protein were electrophoresed in 12.5% or 6% SDS-polyacrylamide gel electrophoresis (PAGE) and then electro-blotted to nitrocellulose membranes. During processing samples from one HI rat were lost. Thus, the final dataset from this component of the study contained 6 LI rats and 5 HI rats.

Immunodetection was performed using:

1. polyclonal rabbit anti-Glial Fibrillary Acidic Protein (GFAP, Dako Cytomation, Denmark), a glial marker;

2. monoclonal mouse anti-Neuronal Nuclei (NeuN, Millipore, USA), a neuron-specific marker;
3. polyclonal rabbit anti-Glutamate Decarboxylase 65/67 ($GAD_{65/67}$, Millipore), the primary GABA synthesizing enzyme;
4. polyclonal rabbit anti-Neurabin II (Spinophilin, Sigma, USA), a dendritic spine marker;
5. monoclonal mouse anti-Microtubule Associated Protein 2 (MAP2, Sigma), a marker for somatodendritic microtubule protein;
6. monoclonal mouse anti-β-Actin (Abcam, UK) a house keeping protein used as a loading control.

Immunoreactivity was detected by a peroxidase-based ECL Detection kit (Amersham, USA). Bands were visualized with a CCD camera (UVP System Ltd., UK) and densitometric quantification was performed with ImageJ (v1.44b, NIH, USA).

Statistical analysis of the Western blot results were carried out using a two tailed Student's *t*-test. Correlations of the results with impulsivity were evaluated by Pearson's product-moment correlation coefficient (r). A William's test was used to evaluate differences between the two dependent rho values (deriving from the same correlation matrix), calculated separately for left and right hemispheres.

Antisense Oligodeoxynucleotides

Fully de-protected and de-salted phosphorothioate oligodeoxynucleotides (ODNs), purified by PAGE, were purchased from Sigma, UK. ODNs were phosphorothioated on the three terminal bases of both 5' and 3' ends, in order to increase stability and minimize non-specific toxicity. The sequences and concentrations of ODNs were derived from previous studies (10,

11): glutamate decarboxylase 67 (GAD_{67}) antisense ODN 5’-TGCCATCAGCTCGGT-3’; GAD_{65} antisense ODN 5’-AGATGCCATGGGTTC-3’; scrambled sequence control for GAD_{67} 5’-GATCGCTAGTCCGTC-3’; scrambled sequence control for GAD_{65} 5’-CTAGCAGTCGATGTG-3’.

Surgery

Rats destined for the GAD antisense experiment were ranked for low impulsivity as described above (n = 23). Anesthesia was induced with isoflurane (5%) and maintained throughout the surgery at 1.5-2% (flow rate, 2 l/min) delivered via a nose cone fitted on the incisor bar of the stereotaxic frame (David Kopf Instruments, Tujunga, CA). The incisor bar was set at -3.3 mm relative to the interaural line (9). Bilateral 22-gauge double-guide cannulae (Plastics One, Sevenoaks, UK) were implanted above the nucleus accumbens core (NAcbC). Stereotaxic coordinates relative to bregma were: AP +1.5, ML ±1.9 and DV -2.2 mm. Guide cannulae were occluded by a stylet and secured to the skull with dental cement and three stainless steel screws. Rats were given two full days in which to recover from surgery.

Intracerebral ODNs Administration

To habituate rats to the intracranial infusion procedure, the stylet was removed each day where it was cleaned (70% ethanol), dried, and re-inserted in the guide cannulae. Infusions were given 8 h before behavioral testing (08:00 h) using a syringe pump (Harvard Apparatus, Holliston, MA) and a 10 µl Hamilton syringe connected via polyethylene tubing to a 28-gauge bilateral injector (Plastic One, Roanoke, USA). Rats remained in their home-cage until behavioral assessment. Injectors aimed at the NAcbC and caudate putamen (CPu) extended 4.5

mm and 2.0 mm, respectively, from the ventral tip of the guide cannulae and were inserted for 30 s before the infusions commenced. Following each infusion injectors were left in place for 60 s to allow diffusion of the ODNs into the NAcbC. Infusions were made over 72 s (0.3 µl per hemisphere), and based on previous study contained 600 ng of either $GAD_{65/67}$ antisense (ASO) or scramble (Scr) pairs (10, 11). We validated the procedure in a separate group of selected low impulsive rats (n = 14), to assess the magnitude of reduction in $GAD_{65/67}$ expression in the NAcbC. We observed a significant reduction of $GAD_{65/67}$ protein levels after infusion of ASO compared to rats injected with Scr ($p < 0.05$) in the NAcbC (Figure 3C). Furthermore, we observed no diffusion into the surrounding NAcb shell region (Fig. 3D) thus confirming the anatomical localization of the NAcbC infusions.

Antisense Behavioral Procedure

Following surgery, LI rats were run on the 5-CSRTT (ITI = 5 s) for 5 consecutive days. They were then challenged with three LITI sessions (ITI = 7 s), each spaced two days apart, to obtain a stable level of premature responding. Rats were then assigned to four groups matched for behavioral performance on the 5-CSRTT ($GAD_{65/67}$ ASO; Scr). The ODNs testing phase consisted of three long ITI sessions (ITI = 7 s) spaced two days apart. On test day 1, all rats received an infusion of phosphate-buffered saline (ODNs vehicle) in the NAcbC. One group received a bilateral infusion of $GAD_{65/67}$ ASO, two groups a unilateral infusion of $GAD_{65/67}$ ASO (left or right - the contralateral site received an infusion of Scr) while the remaining group received Scr bilaterally in the NAcbC. On test day 3, all rats that received a bilateral infusion of ASO or Scr were further infused with phosphate-buffered saline to assess behavioral recovery. The remaining groups received bilateral infusions of $GAD_{65/67}$ ASO or Scr in the CPu.

Histological Assessment of Cannulae Placement

At the completion of the experiment, rats were sacrificed with an i.p. injection of sodium pentobarbital (2 ml per rat, Dolethal 200 mg/ml, Rhone Merieux, Athens, USA) and perfused transcardially with 0.01 M PBS followed by 4% paraformaldehyde. Brains were removed and kept for 24 h in 4% paraformaldehyde followed by 24 h in a 20% sucrose solution in 0.01 M phosphate-buffered saline. Brains were then sliced in 60 µm coronal sections using a cooled microtome. Every third section was mounted and stained with Cresyl-Violet. Cannulae placements were verified under a light microscope and mapped onto published coronal sections of the rat brain (9).

Table S1. Behavior of LI, MI and HI rats on the 5-choice serial reaction time task.

	**LI (*n* = 6)**	**MI (*n* = 6)**	**HI (*n* = 6)**
**Baseline**			
% premature responses	5.97 ± 0.59	5.81 ± 0.52	9.87 ± 0.96 (**)(††)
% choice accuracy	85.0 ± 0.39	83.02 ± 0.50	77.76 ± 0.66
% omissions	5.67 ± 0.44	6.21 ± 0.29	7.66 ± 0.51
latency to respond (ms)	617.13 ± 8.34	705.18 ± 13.90	693.15 ± 9.92
magazine latency (ms)	1664.63 ± 12.60	1510 ± 13.05	1268.16 ± 10.67
**LITI sessions**			
% premature responses	24.67 ± 3.38	44.88 ± 2.59 (#)	76.55 ± 5.70 (***)(††)
% choice accuracy	84.02 ± 1.13	77.14 ± 1.90	66.30 ± 1.53 (**)(†)
% omissions	6.39 ± 0.39	12.10 ± 1.42	18.20 ± 1.87
latency to respond (ms)	561.83 ± 25.25	585. 53 ± 4.84	645.48 ± 7.80
magazine latency (ms)	1613.73 ± 16.26	1451.57 ± 64.77	1206.01 ± 21.88

LI, low-impulsive; MI, mid-impulsive; HI, high-impulsive; ITI, intertrial interval.

Data shown are means ± SEM for twelve consecutive sessions on the 5-CSRTT (ITI = 5 s) and across three long ITI sessions (LITI = 7 s).

Differences between HI and LI rats are indicated as $^{**}p < 0.01$, $^{***}p < 0.001$.

Differences between HI and MI rats are indicated as $^{\dagger}p < 0.05$, $^{\dagger\dagger}p < 0.01$.

Differences between MI and LI rats are indicated as $^{\#}p < 0.05$.

Table S2. Behavioral effects of intra-NAcb core $GAD_{65/67}$ ODNs infusions

	Screening	Veh-pre		$GAD_{65/67}$-ODNs		Veh-post	
	(*n* = 13)	ASO (*n* = 7)	Scr. (*n* = 6)	ASO (*n* = 7)	Scr. (*n* = 6)	ASO (*n* = 7)	Scr. (*n* = 6)
**Baseline**		**A**		**C**		**E**	
% premature responses	7.6 ± 0.9	5.5 ± 1.1	5.6 ± 1.0	8.4 ± 1.7	6.7 ± 1.3	5.7 ± 1.8	5.1 ± 1.3
% choice accuracy	81.7 ± 1.5	84.8 ± 1.6	87.2 ± 2.6	85.2 ± 2.4	85.7 ± 2.5	86.9 ± 1.9	85.9 ± 2.9
% omissions	12.1 ± 2.1	10.1 ± 3.1	8.7 ± 3.4	10.7 ± 3.6	9.2 ± 2.0	10.7 ± 4.8	12.9 ± 3.6
latency to respond (ms)	603.8 ± 35.0	499.1 ± 29.1	511.3 ± 30.4	531.7 ± 41.3	528.9 ± 37.5	541.9 ± 35.2	565.0 ± 28.9
magazine latency (ms)	1836.3 ± 171.1	1670.6 ± 202.0	1586.7 ± 194.3	1879.7 ± 182.2	1701.6 ± 170.7	1843.3 ± 166.6	1567.6 ± 131.7
**LITI sessions**		**B**		**D**		**F**	
% premature responses	27.6 ± 1.8	25.4 ± 4.2	27.5 ± 3.2	46.3 ± 8.3$^{(*)(\dagger)}$	32.2 ± 5.2	28.3 ± 5.2	23.0 ± 6.0
% choice accuracy	82.3 ± 1.5	84.3 ± 2.5	87.5 ± 2.7	80.6 ± 2.9	88.3 ± 2.3$^{(\dagger)}$	80.7 ± 4.3	82.4 ± 1.8
% omissions	15.1 ± 3.3	11.8 ± 3.6	7.3 ± 2.0	10.9 ± 3.6	11.5 ± 2.3	10.0 ± 4.3	17.1 ± 7.6
latency to respond (ms)	538.4 ± 28.9	495.4 ± 32.3	497.9 ± 18.6	482.0 ± 33.3	498.2 ± 21.5	489.3 ± 32.5	587.3 ± 44.8
magazine latency (ms)	1829.1 ± 108.3	2416.3 ± 507.9	1548.7 ± 163.6	1795.3 ± 215.3	1432.6 ± 130.4	1691.3 ± 287.0	1635.7 ± 220.3

GAD, glutamate decarboxylase; ITI, intertrial interval; NAcb, nucleus accumbens; ODNs, oligodeoxynucleotides; Veh, vehicle.

The first column refers to the screening procedure for twelve consecutive sessions on the 5-choice serial reaction time task (ITI = 5 s) and across three long ITI sessions (LITI = 7 s). The upper half of the table (A,C,E) refers to two consecutive baseline sessions before the ODN infusions while the lower half of the table refers to long ITI sessions (D,E,F) where antisense (ASO) or scramble (Scr) was administered. Data shown are means ± SEM.

Differences between vehicle pre-treated and ODN-treated are reported respectively for the ASO and Scr groups and indicated as $*p < 0.05$.

Differences between ODNs-treated and vehicle post-treatment are reported as $^{\dagger}p < 0.05$.

Table S3. Behavioral effects of unilateral (left or right) intra-NAcb core $GAD_{65/67}$ ASO infusions and bilateral intra-CPu $GAD_{65/67}$ ODNs infusions.

	Screening (*n* = 10)	NAcb core		NAcb core		CPu	
		Veh-pre (1)	Veh-pre (2)	left ASO (1)	right ASO (2)	ASO (1)	Scr. (2)
**Baseline**		A		C		E	
% premature responses	8.7 ± 0.9	7.0 ± 1.7	10.0 ± 2.6	8.3 ± 1.2	9.0 ± 2.1	6.2 ± 0.9	7.8 ± 1.0
% choice accuracy	85.7 ± 0.9	89.1 ± 1.7	84.9 ± 1.6	87.8 ± 3.7	81.5 ± 3.3	88.4 ± 2.2	83.3 ± 2.3
% omissions	10.8 ± 1.8	9.1 ± 2.3	6.6 ± 2.3	12.7 ± 3.7	7.7 ± 3.0	7.0 ± 1.3	8.3 ± 2.5
latency to respond (ms)	521.6 ± 28.4	484.3 ± 38.5	550.6 ± 48.8	488.9 ± 33.9	562.4 ± 52.3	505.5 ± 52.4	575.3 ± 50.7
magazine latency (ms)	2490.9 ± 681.8	1346.4 ± 47.7	1620.9 ± 162.6	1492.4 ± 69.3	1671.9 ± 86.7	1354.1 ± 66.1	1980.1 ± 235.5
**LITI sessions**		B		D		F	
% premature responses	28.7 ± 1.6	27.8 ± 3.4	26.2 ± 3.5	33.2 ± 2.8	29.2 ± 5.5	28.8 ± 2.5	31.6 ± 4.9
% choice accuracy	85.9 ± 1.2	87.2 ± 2.0	81.6 ± 2.6	88.7 ± 2.4	82.8 ± 2.8	88.2 ± 1.5	81.8 ± 2.3
% omissions	10.0 ± 2.4	12.0 ± 4.2	12.2 ± 5.0	5.0 ± 1.5	8.8 ± 3.4	13.0 ± 4.3	7.8 ± 2.9
latency to respond (ms)	501.7 ± 25.4	570.2 ± 124.7	561.0 ± 49.8	456.5 ± 28.8	496.9 ± 31.4	449.7 ± 26.9	529.7 ± 49.2
Magazine latency (ms)	2085.5 ± 297.4	1509.0 ± 123.22	1816.5 ± 176.4	1373.8 ± 60.6	1901.1 ± 333.6	1487.6 ± 150.5	1788.6 ± 169.6

CPu, caudate putamen; GAD, glutamate decarboxylase; ITI, intertrial interval; NAcb, nucleus accumbens; ODNs, oligodeoxynucleotides; Veh, vehicle.

The first column refers to the screening procedure for twelve consecutive sessions on the 5-choice serial reaction time task (ITI = 5 s) and across three long ITI sessions (LITI = 7 s). The upper half of the table (A,C,E) refers to two consecutive baseline sessions before the ODN infusions while the lower half of the table refers to long ITI sessions (D,E,F) where antisense (ASO) or scramble (Scr) was administered. Data shown are means ± SEM.

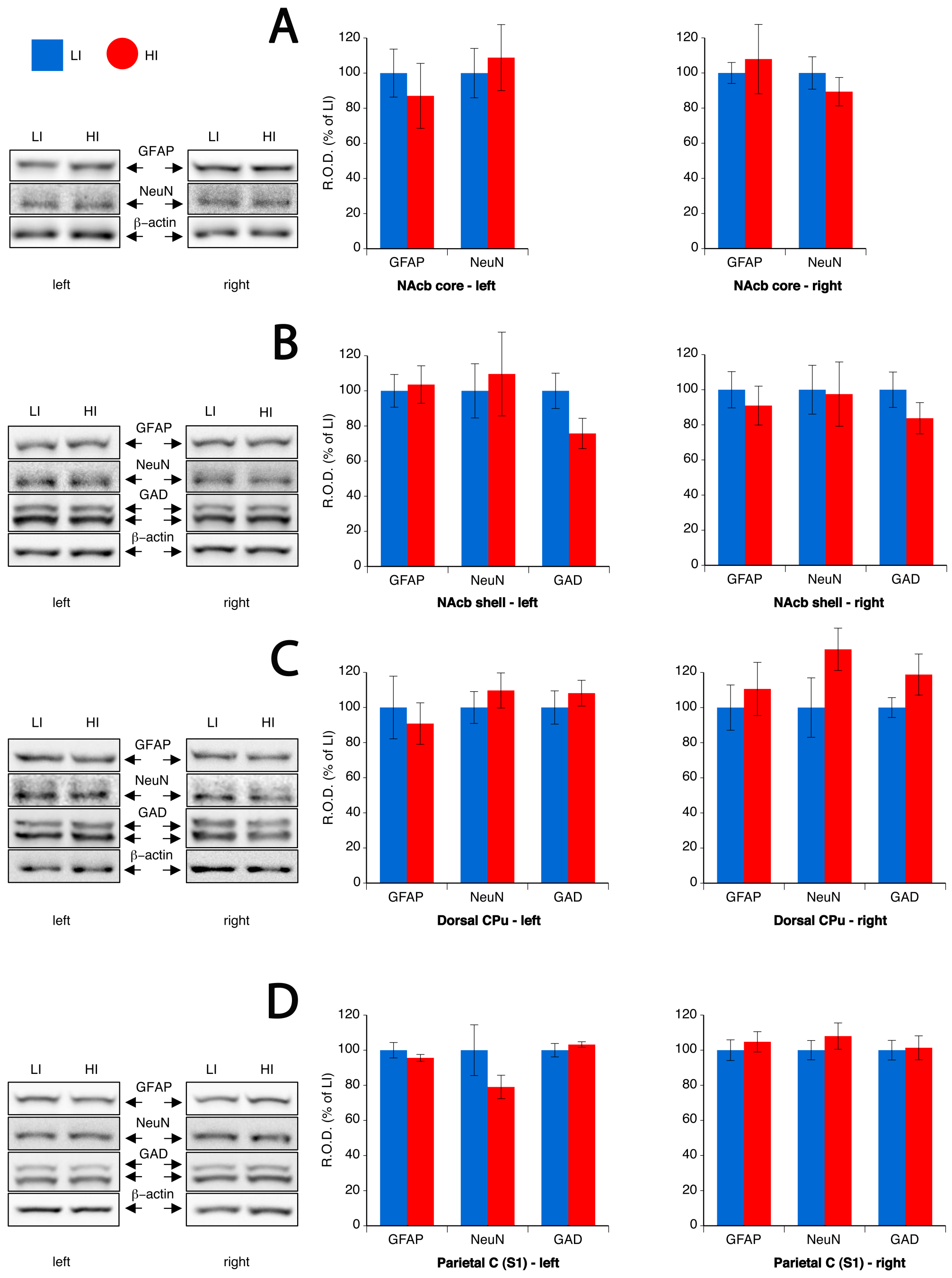

Figure S1. Western blot analysis of regional protein expression in the left and right nucleus accumbens core and shell (NAcbC and NAcbS), dorsal caudate putamen (CPu) and parietal cortex (S1) of LI and HI rats. Representative immunoreactive bands and relevant densitometric analyses (bar plots) are shown. No significant differences in GFAP and NeuN were found in the NAcbC of HI and LI rats (**A**), or between GFAP, NeuN and GAD 65/67 in the NAcbS (**B**), dorsal CPu (**C**) or fronto-parietal cortex (**D**). All Western blots were normalized against β-actin to control for protein loading, and are expressed as a percentage of LI rats. GFAP, glial fibrillary acidic protein; LI, low-impulsive; HI, high-impulsive; NeuN, neuronal nuclei; R.O.D., relative optic density.

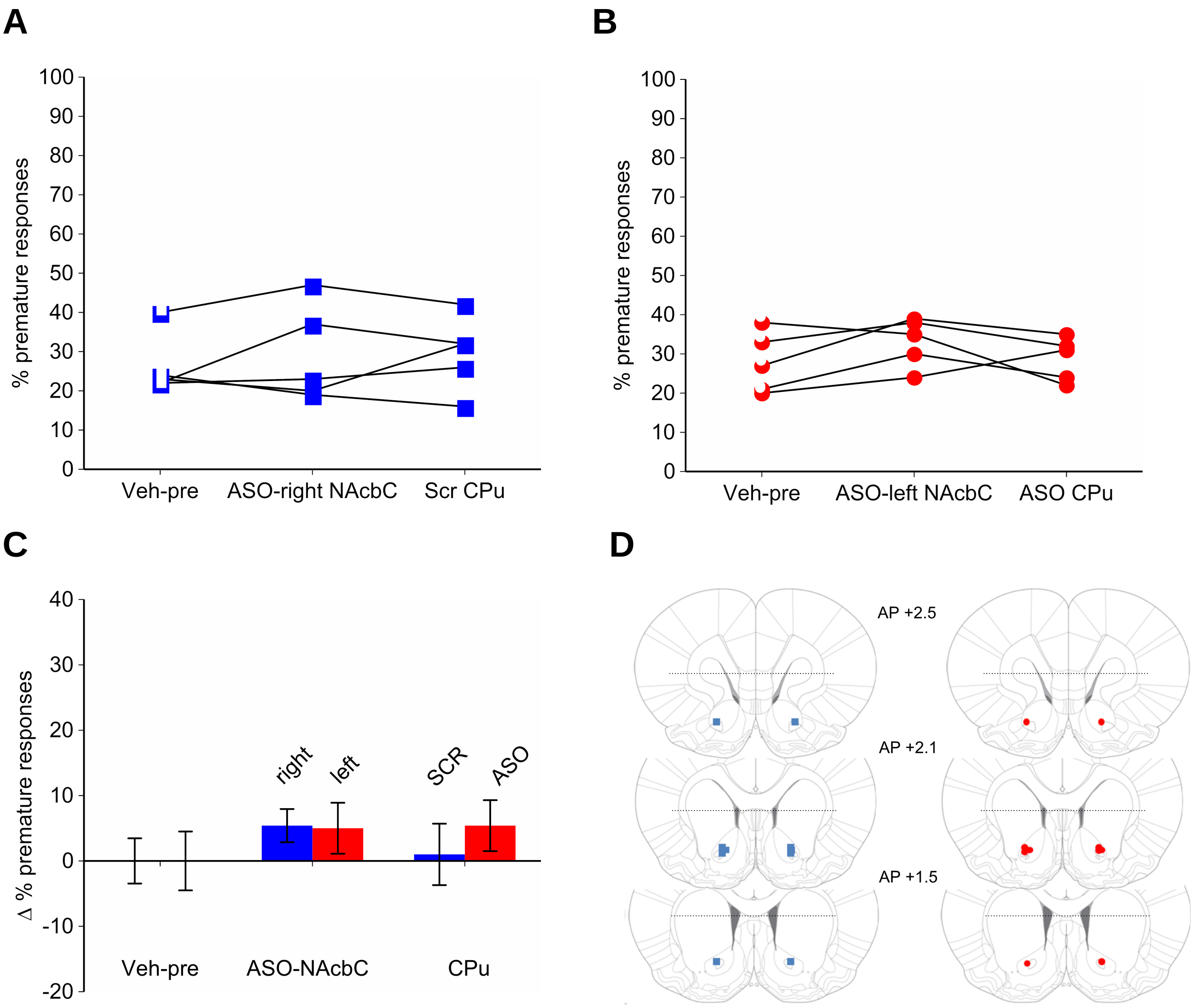

Figure S2. Effects of unilateral and bilateral reduction in $GAD_{65/67}$ protein respectively in the nucleus accumbens core (NAcbC) and caudate-putamen (CPu) on 5-choice serial reaction time task behavioral performance. (**A**) Individual responses of rats to right-antisense (ASO) and left-scramble (Scr.) microinfusions in the NAcbC showing no effect on premature responding compared with vehicle infusions in this region (n = 5). We next injected $GAD_{65/67}$ Scr. bilaterally in the CPu. This had no significant effect on impulsivity. (**B**) Individual responses of rats to left-ASO and right-Scr. microinfusions in the NAcbC showing no effect on premature responding compared with vehicle infusions in this region (n = 5). We next injected $GAD_{65/67}$ ASO bilaterally in the CPu. This intervention had no significant effect on impulsivity. (**C**) Histograms show difference scores (± SEM) between the effects of vehicle infusions [pre- and post-oligodeoxynucleotide (ODNs)] and ODNs infusions. (**D**) Injector tip locations in the NAcbC of rats injected with $GAD_{65/67}$ ASO in the left hemisphere (right panel) and right hemisphere (left panel). Anterior-posterior (AP) coordinates are relative to bregma (mm). The dashed lines refer to the intended injector tip locations in the CPu. Reprinted with permission from (9).

Supplemental References

1. Bari A, Dalley JW, Robbins TW (2008): The application of the 5-choice serial reaction time task for the assessment of visual attentional processes and impulse control in rats. *Nat Protoc*. 3:759-767.

2. Cardinal RN, Aitken MR (2010): Whisker: a client-server high-performance multimedia research control system. *Behav Res Methods*. 42:1059-1071.

3. Sawiak SJ, Wood NI, Williams GB, Morton AJ, Carpenter TA (2009): Voxel-based morphometry in the R6/2 transgenic mouse reveals differences between genotypes not seen with manual 2D morphometry. *Neurobiol Dis*. 33:20-27.

4. Ashburner J, Friston KJ (2000): Voxel-based morphometry--the methods. *Neuroimage*. 11:805-821.

5. Sawiak SJ, Williams GB, Wood NI, Morton AJ, Carpenter TA (2009): SPMMouse: A New Toolbox for SPM in the animal brain. *ISMRM 17th Scientific Meeting*. Honolulu, Hawaii, pp 1086.

6. MacKay DJC (2003): *Information theory, inference and learning algorithms*. Cambridge: Cambridge University Press.

7. Ashburner J, Friston KJ (2005): Unified segmentation. *Neuroimage*. 26:839-851.

8. Genovese CR, Lazar NA, Nichols T (2002): Thresholding of statistical maps in functional neuroimaging using the false discovery rate. *Neuroimage*. 15:870-878.

9. Paxinos G, Watson C (2007): *The rat brain in stereotaxic coordinates*. In: Elsevier Academic Press A, editor.

10. Miner LA, Sarter M (1999): Intra-accumbens infusions of antisense oligodeoxynucleotides to one isoform of glutamic acid decarboxylase mRNA, GAD65, but not to GAD67 mRNA, impairs sustained attention performance in the rat. *Brain Res Cogn Brain Res*. 7:269-283.

11. McCarthy MM, Masters DB, Rimvall K, Schwartz-Giblin S, Pfaff DW (1994): Intracerebral administration of antisense oligodeoxynucleotides to GAD65 and GAD67 mRNAs modulate reproductive behavior in the female rat. *Brain Res*. 636:209-220.
